# On the importance of accurate elasto-plastic material properties in simulating plate osteosynthesis failure

**DOI:** 10.3389/fbioe.2023.1268787

**Published:** 2023-12-01

**Authors:** Dominic Mischler, Boyko Gueorguiev, Markus Windolf, Peter Varga

**Affiliations:** ^1^ AO Research Institute Davos, Davos, Switzerland; ^2^ Graduate School for Cellular and Biomedical Sciences (GCB), University of Bern, Bern, Switzerland

**Keywords:** osteosynthesis, finite element simulation, plasticity, plate bending, tensile testing

## Abstract

**Background:** Plate osteosynthesis is a widely used technique for bone fracture fixation; however, complications such as plate bending remain a significant clinical concern. A better understanding of the failure mechanisms behind plate osteosynthesis is crucial for improving treatment outcomes. This study aimed to develop finite element (FE) models to predict plate bending failure and validate these against *in vitro* experiments using literature-based and experimentally determined implant material properties.

**Methods:** Plate fixations of seven cadaveric tibia shaft fractures were tested to failure in a biomechanical setup with various implant configurations. FE models of the bone-implant constructs were developed from computed tomography (CT) scans. Elasto-plastic implant material properties were assigned using either literature data or the experimentally derived data. The predictive capability of these two FE modelling approaches was assessed based on the experimental ground truth.

**Results:** The FE simulations provided quantitatively correct prediction of the *in vitro* cadaveric experiments in terms of construct stiffness [concordance correlation coefficient (CCC) = 0.97, standard error of estimate (SEE) = 23.66, relative standard error (RSE) = 10.3%], yield load (CCC = 0.97, SEE = 41.21N, RSE = 7.7%), and maximum force (CCC = 0.96, SEE = 35.04, RSE = 9.3%), when including the experimentally determined material properties. Literature-based properties led to inferior accuracies for both stiffness (CCC = 0.92, SEE = 27.62, RSE = 19.6%), yield load (CCC = 0.83, SEE = 46.53N, RSE = 21.4%), and maximum force (CCC = 0.86, SEE = 57.71, RSE = 14.4%).

**Conclusion:** The validated FE model allows for accurate prediction of plate osteosynthesis construct behaviour beyond the elastic regime but only when using experimentally determined implant material properties. Literature-based material properties led to inferior predictability. These validated models have the potential to be utilized for assessing the loads leading to plastic deformation *in vivo*, as well as aiding in preoperative planning and postoperative rehabilitation protocols.

## 1 Introduction

**FIGURE 1:**
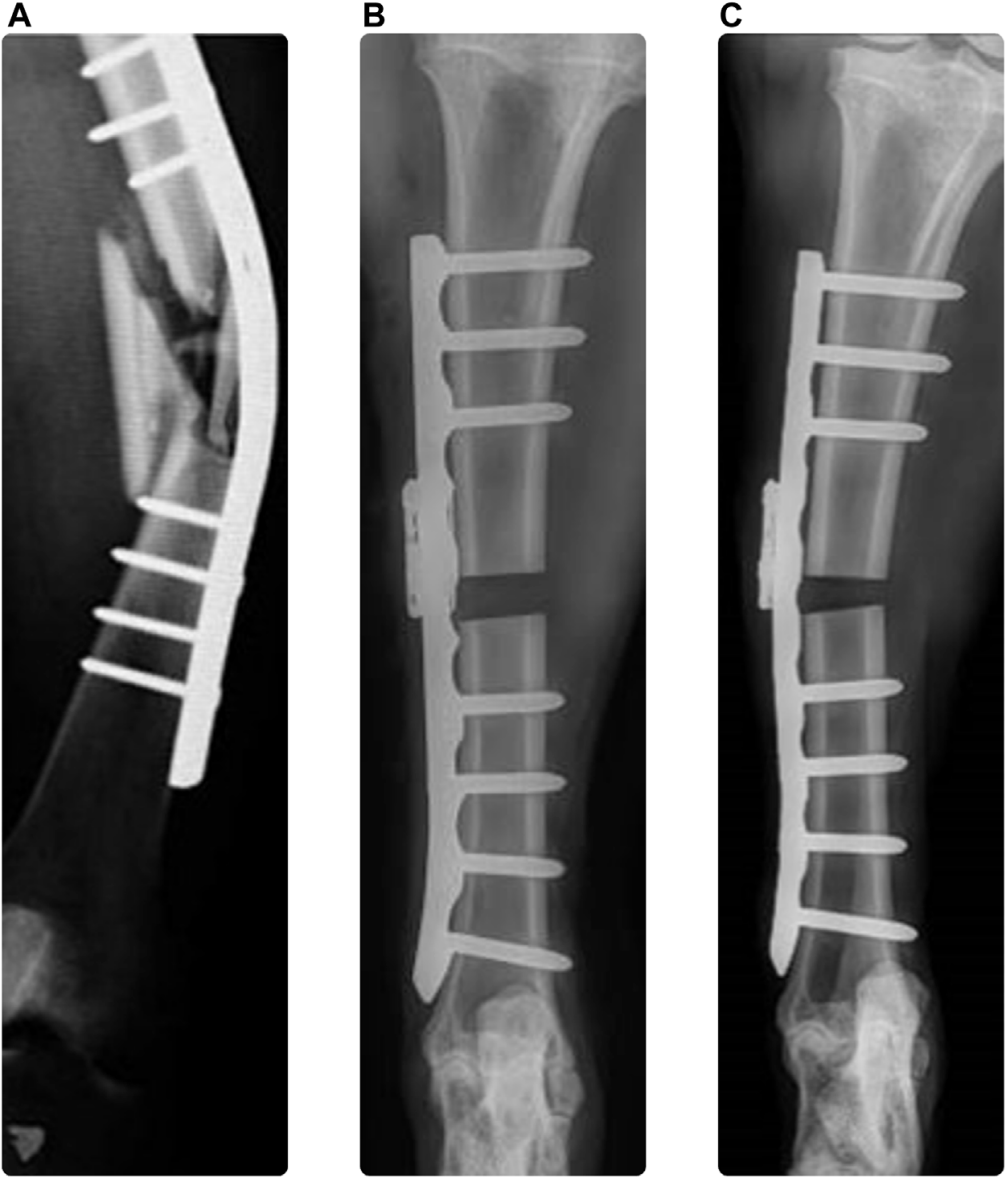
Clinical case demonstrating plate bending [**(A)** (Gueorguiev and Lenz, 2018)], immediate post operative x-ray of ovine tibia [**(B)** (Windolf et al., 2022)], and 1 week follow up with plastic plate bending [**(C)** (Windolf et al., 2022)].

Plate osteosynthesis is a well-established technique applied for fixation of bone fractures with use of metallic plates and screws to stabilize the reduced fracture, promote bone healing, and restore function (Perren, 2002). Trauma fixation devices are implemented to treat approximately 5%–10% of all bone fractures, corresponding to 320,000 and 1.1 million cases in Germany and the United States, respectively (GlobalData_MediPoint, 2016). Approximately 90% of these devices are internal fixators. Despite the impressive evolution of existing implants and guidelines throughout the recent decades (Perren, 2002; Augat and von Ruden, 2018), failure rates of up to 18% have been reported for plating of long bone fractures over the observed follow-up periods of approximately 12 months (Banovetz et al., 1996; Button et al., 2004; Kregor et al., 2004; Vallier et al., 2006), usually requiring reoperation. Additionally, non-unions occur in 5%–10% of the cases (Zura et al., 2016; Ekegren et al., 2018), increasing the burden on both the patients’ quality of life and the healthcare system (Hak et al., 2014; Rupp et al., 2018). Mechanics plays a fundamental role in the occurrence of fixation failures and healing complications (Sommer et al., 2004; Rizk and Al-Ashhab, 2015). Insufficient construct stability can lead to loss of reduction or to failure of the implant, the bone, or their interface. Implant malfunction or failure is one reason contributing to the high complication rate with up to 29% of the cases (Korner et al., 2005; Koso et al., 2018; Yetter et al., 2021), suggesting the need for improvements in the design and use of the implants (Gueorguiev and Lenz, 2018). Overloading failure may occur in unsuitably treated cases, where the fracture reduction, selection and configuration of the implants are inadequate and lead to insufficient construct strength, even in correctly performed fixations via too early and aggressive weightbearing due to the recommended rehabilitation protocol or poor patient compliance (Figure 1A). This risk may be substantial, especially in case of load bearing fixations such as bridge plating of the tibia or femur. Similarly, in a recent preclinical study, plate bending was observed in plated ovine tibia shaft fractures (Figure 1C) compared to the direct post-operative state (Figure 1B). Plate deformation could be observed radiologically already 1 week post operatively, suggesting early implant overloading.

Finite element (FE) modelling has emerged as a valuable tool for prediction of construct behaviour in various fields, including traumatology, orthopaedics and biomechanics (Taylor and Prendergast, 2015), providing valuable insights into the biomechanical performance of various implant designs and configurations (Mischler et al., 2020). Although validated FE modelling has been proven to be effective in predicting construct behaviour, most studies have relied on literature-based material properties and ignored post-elastic behavior, which can introduce uncertainties and limit the accuracy of the predictions (Lewis et al., 2021). This indicates the need for experimentally determined material properties to ensure accurate and reliable simulations of osteosynthesis constructs’ behaviour.

Towards the overarching goal to describe *in vivo* implant failures, the first step is to understand the construct behaviour in a more controlled *in vitro* setting. Therefore, the main aim of this study was to investigate the elastic and plastic biomechanical behaviour of plate fixation constructs using experimental testing and to establish validated specimen-specific FE models predicting construct stiffness, yield and maximum load. A secondary aim was to compare the predictive ability of the FE models using either experimentally determined elastic and plastic implant material properties or literature-based properties.

It was hypothesized that the FE models can more accurately predict fixation construct behaviour with use of experimentally determined elastic and plastic implant material properties versus use of literature-based properties.

## 2 Materials and methods

### 2.1 Study overview

**FIGURE 2:**
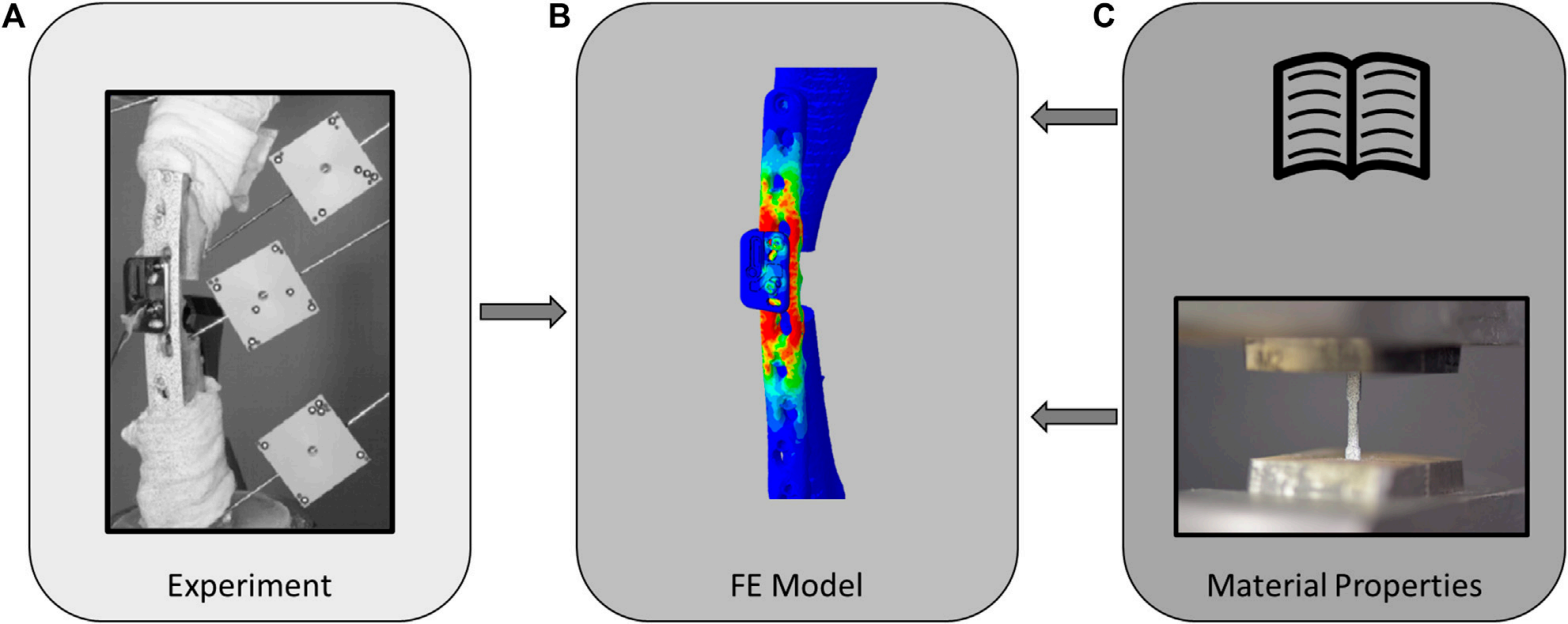
Schematic of the study methodology involving *in vitro* cadaveric experiments **(A)** which were used to validate the elasto-plastic finite element models **(B)**, using either literature-based [**(C)**, top] or experimentally determined [**(C)**, bottom] material properties.

Osteotomized ovine tibiae fixed with locking plates were biomechanically tested (Figure 2A). Subject-specific FE models of the fracture fixation constructs were then built based on preoperative and postoperative computed tomography (CT) images (Figure 2B). Elastic and plastic material properties of the osteosynthesis implants were either determined from literature data (Mat_Lit_) or measured experimentally via uniaxial tensile testing (Mat_Exp_) and integrated into the models (Figure 2C). FE simulations with elasto-plastic material behaviour replicated the biomechanical conditions and the models’ predictions were compared with the experimental results to assess accuracy and reliability. The fixations used in this study replicated a previous preclinical study, including the attachment of the AO Fracture Monitor (Windolf et al., 2022), to ensure relevance of these procedures for future investigation of the *in vivo* situation. These steps are described in more detail in the following sections.

### 2.2 Biomechanical testing

**FIGURE 3:**
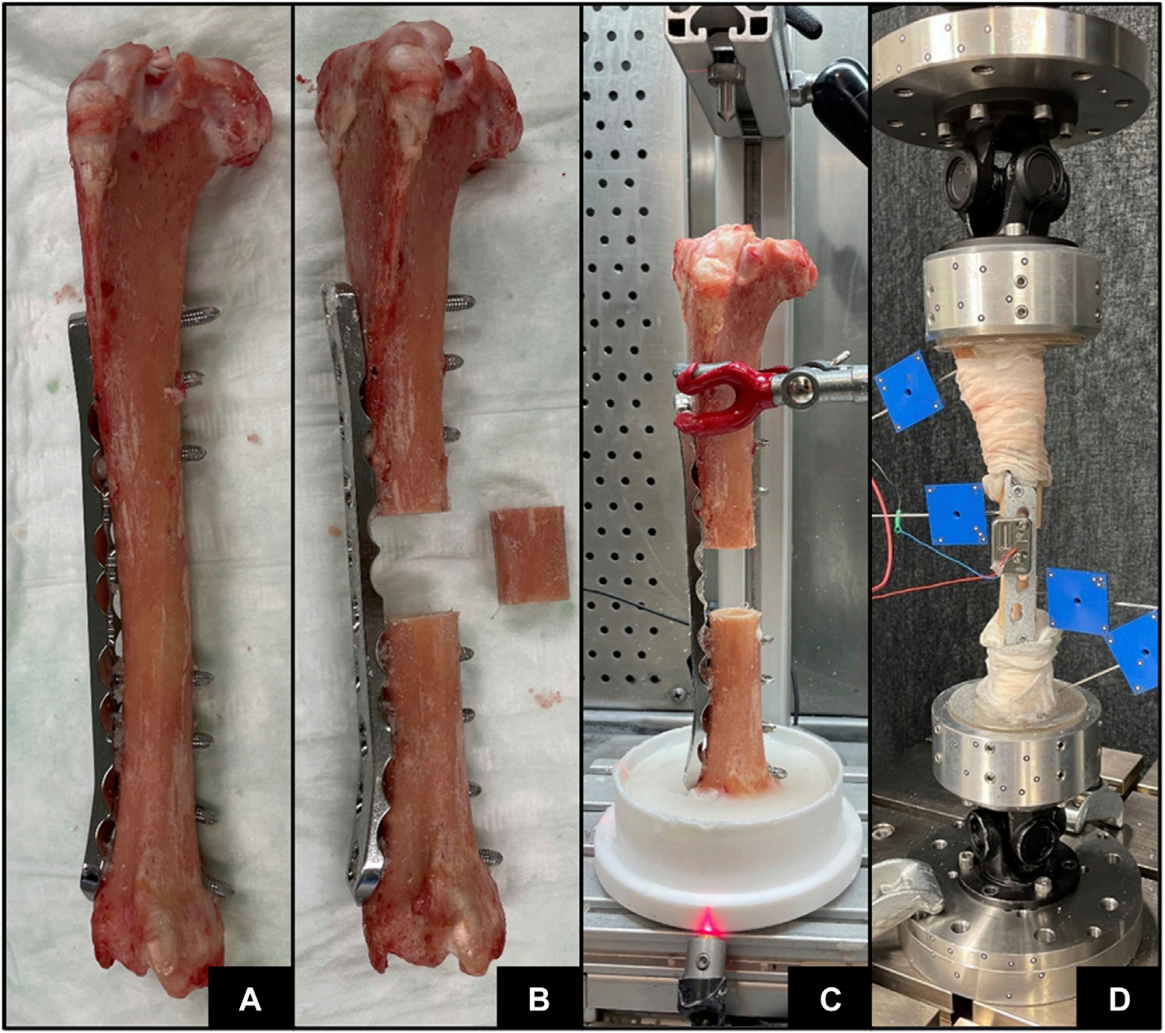
Specimen preparation steps with implant instrumentation **(A)**, osteotomy **(B)**, PMMA embedding **(C)**, and subsequent biomechanical testing **(D)**.

Seven fresh-frozen intact sheep tibiae were stripped from soft tissue and thawed for 6 hours at room temperature. The bones were taken from previous studies; thus, no animals were sacrificed for this investigation. Preoperative clinical CT (Revolution EVO, GE Healthcare, Chicago, IL, United States) scans of the intact tibiae were acquired with scanning settings of 120 kV voltage, 200 mA current and 0.625 mm slice thickness, and calibrated to volumetric bone mineral density (vBMD) units using a density phantom (QRM-BDC/6, QRM GmbH, Moehrendorf, Germany). Five specimens were instrumented with a veterinary stainless steel locking compression plate (LCP) (5.5 mm, broad, 10 holes, DePuy Synthes, Zuchwil, Switzerland) and two with human titanium LCP (4.5/5.0 mm, broad, 12 holes, DePuy Synthes, Zuchwil, Switzerland) creating a relative stability construct according to the bridge plating principle (Figure 3A). After instrumentation, different osteotomy sizes were created using a surgical oscillating saw with a 0.8 mm thick blade (DePuy Synthes) in combination with cutting guides to ensure alignment of the cuts (Figure 3B; Table 1). The epiphyseal regions of the instrumented tibiae were embedded in polymethylmethacrylate (PMMA, SCS-Beracryl; Suter-Kunststoffe AG, Fraubrunnen, Switzerland) using a laser-guided embedding station (Figure 3C) to ensure axial alignment. Prior to mounting for biomechanical testing, CT scanning of the constructs was performed with the same device and settings described above to capture the postoperative state including embedding. Following instrumentation, the constructs were mounted on a testing machine (Instron 5866, Norwood, MA, United States) using cardan joints proximally and distally (Figure 3D). The specimens were axially loaded to a maximum actuator displacement of 14 mm until plastic deformation of the implant was reached, i.e., after plateauing and consequent decrease of the force-displacement curve, and subsequently unloaded to 0 N at a displacement rate of 0.04 mm/s. The displacements and rotations of the bone fragments and the test setup components were recorded at 5 Hz using a stereographic high-resolution motion tracking camera system (Aramis SRX, GOM GmbH, Braunschweig, Germany) and custom marker sets (Figure 3D). A setup-specific coordinate system was defined, originating in the distal cardan joint, with the x-axis being the machine loading axis and the y-axis oriented normal to the upper plane of the LCP. Construct displacement without potential machine setup compliance was determined via landmarks at the proximal and distal cardan joints which were defined using a touch probe and virtually coupled to the bone fragment markers. Axial load was measured using a 3 kN load cell mounted proximally to the upper cardan joint. Additionally, four landmarks at the near and far cortices directly adjacent to the osteotomy, and two points at the distal and proximal ends of the specimen—where the embedding plane intersected the loading axis—were digitized using the touch probe of the camera system for alignment of the FE model. Similarly, the resulting experimental displacement was used in the FE simulation as displacement input.

**TABLE 1:** Construct configurations of the tested specimens. Distance between the screw holes equates to 18 mm.

Specimen	Implant material	Osteotomy gap size [mm]	Screw configuration (from proximal to distal)
S1	Stainless steel	32.0	1110000111
S2	Stainless steel	31.2	1110001111
S3	Stainless steel	83.7	1100000011
S4	Stainless steel	28.7	1110001111
S5	Stainless steel	30.0	1110000111
T1	Titanium	30.3	001110001111
T2	Titanium	59.6	001110000111

### 2.3 Implant material property assessment

**FIGURE 4:**
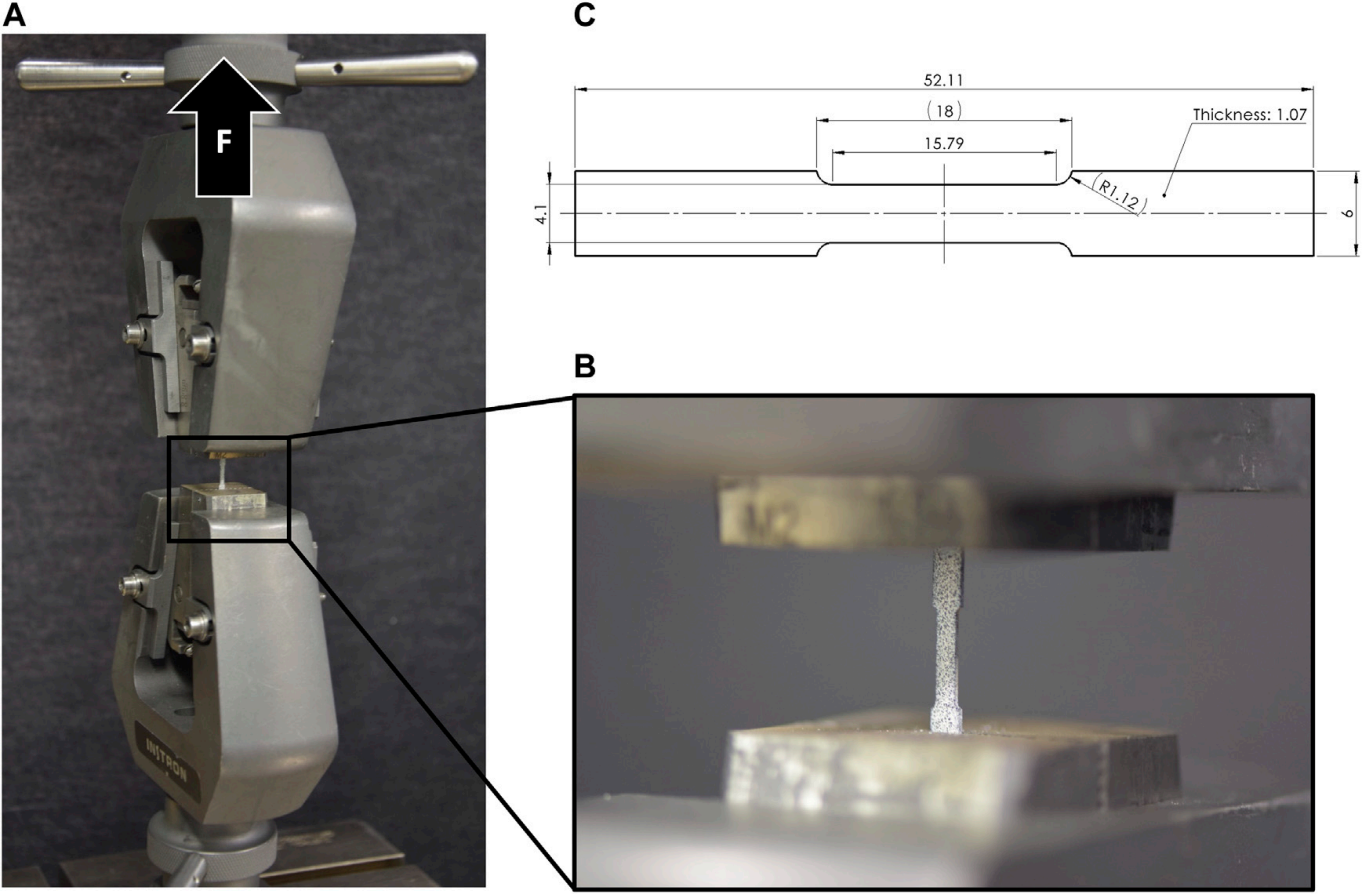
Tensile testing setup **(A)** with clamped dog bone sample **(B)**, and specimen dimensions **(C)** with scaled-down dimensions of the ASTM D638 tensile test protocol (ASTM International, 2014).

Elastic and plastic material properties of implant-grade stainless steel (*n* = 10, 1.4441, Robert Mathys Co., Bettlach, Switzerland) and titanium (*n* = 11, Ti-Grade 4, 3.7065, Robert Mathys Co., Bettlach, Switzerland) were determined by uniaxial tensile tests using a material testing machine (Instron 5866, Norwood, MA, United States). Dog bone shaped samples were harvested from raw material plate profiles scaled to maximum possible dimensions (Figure 4). The samples were mounted using special clamps and loaded in tension at a constant displacement rate of 0.04 mm/s until failure. The reaction force was measured using a 10 kN load cell. Each sample was covered with a speckle pattern and surface deformation was measured using an optical camera system (Aramis SRX) by tracking two virtual surface points defined at a constant gauge length of 15.79 mm in the midsection on the sample. Strain was evaluated as the relative displacement of these points divided by the original gauge length. Engineering stress was determined based on the initial cross-sectional area of the test samples without considering necking that occurred during the tests. Youngs modulus was determined from the initial slope of the stress-strain curve, yield point was defined using an 0.2% offset approach (Rp 0.2), and ultimate elongation at failure of the sample was measured.

### 2.4 Subject-specific FE modelling and validation

**FIGURE 5:**
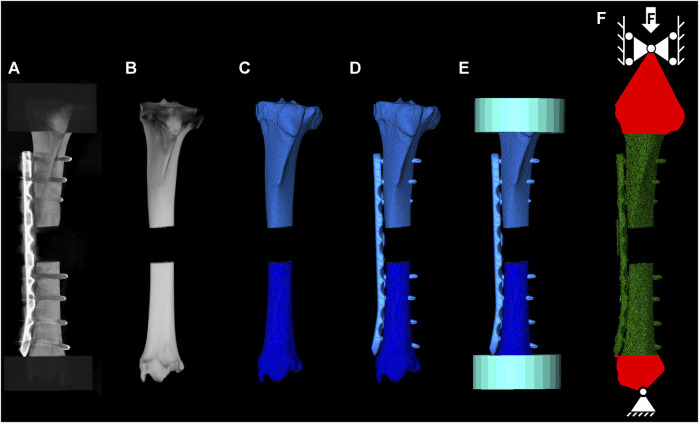
FE model building workflow. Instrumented CT scan used to quantify the post operative construct state **(A)**, co-registered intact CT scan **(B)**, segmented mask for the proximal and distal bone **(C)**, segmented and co-registered implant for CAD file positioning **(D)**, co-registered cylinders to define the PMMA embedding region **(E)**, and meshed construct and applied boundary conditions reflecting the experimental setup **(F)**.

All seven *in vitro* osteosynthesis constructs were virtually replicated based on the intact and postoperative CT scans for accurate quantification of bone material properties and implant position, respectively (Figure 5). First, the two bone fragments were segmented from the intact scan and co-registered with the postoperative scan. Then, the osteotomy was replicated, and the fragments were transformed to match the alignment in the experimental test setup based on the two landmarks defined on the proximal and distal end of the bone, as well as the four landmarks on the near and far cortices at the osteotomy gap. Computer-aided designs (CAD) of the plate and screws were spatially co-registered with their postoperative CT-based locations using Amira 3D software (v2021, Thermo Fisher Scientific, Waltham, United States). The models were meshed with 398,419 ± 66,647 quadratic tetrahedral elements (C3D10) using Simpleware ScanIP (Synopsys, Mountain View, CA, United States). The appropriate element size was determined in a mesh convergence sub-study. Elastic moduli of bone elements were mapped based on the BMD values of the preoperative CT scan using an established conversion law (Dragomir-Daescu et al., 2011) that was co-registered with the proximal and distal fragment individually. Elastic and plastic implant material properties were assigned based on two different approaches. In the first approach, the properties were taken from previous literature (Mat_Lit_) (Chen et al., 2010; Zhou et al., 2017; Antoniac et al., 2019; Pengrung et al., 2019; Beirami et al., 2021; Mohandes et al., 2021; Takahashi et al., 2021). In the second approach, the experimentally determined results were used (Mat_Exp_) based on the averaged stress-strain curve of all samples of a given material. Engineering strain of the test data was converted to true strain and an elasto-plastic behaviour was defined with Young’s modulus, yield stress, ultimate stress, and the plastic stress-strain behaviour. Poisson’s ratio was defined as 0.35 for both implant materials and as 0.3 for bone. Tie constraints were applied at the bone-screw and the screw-plate interfaces. Boundary conditions were defined to replicate the experimental setup as follows. The PMMA embedding was not included in the models. Instead, reference nodes located at the centers of the proximal and distal cardan joints were kinematically coupled with the PMMA-embedded bone surfaces, and the experimentally measured displacements of the cardan joints were applied to these nodes. To ensure that the loading axis of the specimen was the same as in the experimental setup, the digitized four landmarks at the osteotomy site and the two markers at the proximal and distal end of the specimen—where the embedding plane intersected the axis between the two cardan joints—were used to align the FE model with the experimental specimen using an Iterative Closest Point (ICP) algorithm. The FE analyses were solved using the implicit solver of Abaqus (v2019, Dassault Systèmes, Vélizy-Villacoublay, France) considering geometrical nonlinearities. Construct stiffness was defined from the initial slope of the force-displacement curve below 300 N. Yield load was determined by shifting the initial slope by 1 mm and identifying the intersection point. Furthermore, the maximum reaction force was evaluated. The FE predictions of all three outcome measures were compared with the corresponding experimental outcomes using linear regression analysis involving all seven specimens and the concordance correlation coefficients (CCC), standard error of estimate (SEE), and relative standard error (RSE, Eq. 1) were calculated.

$$RSE\left[\%\right]=100*\sqrt{\sum_{i=1}^{n}\frac{{\left[\frac{x_{i}^{\prime }-x_{i}}{x_{i}}\right]}^{2}}{n-p}}$$ (1)

where *n* is the number of specimens, *p* the type of the curve (*p* = 2, as a linear curve was used), $x_{i}$ the experimental data (ground truth), and $x_{i}^{\prime }$ the FE data.

## 3 Results

### 3.1 Biomechanical evaluation of axial loading on plate bending

**FIGURE 6:**
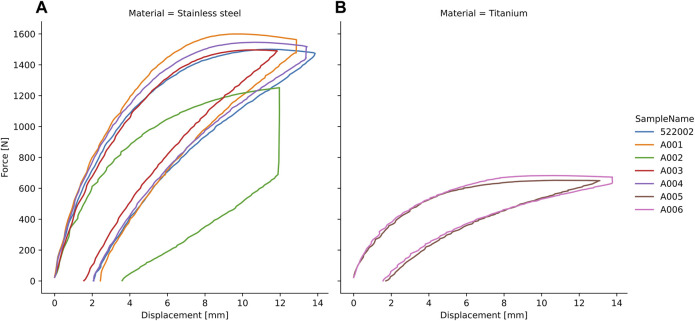
Force-displacement curves of the ovine tibiae instrumented with stainless steel **(A)** and titanium **(B)** plates and experimentally tested under axial compression until plastic deformation with consequent unloading.

The cadaveric experiments induced overloading failure of the constructs resulting in permanent deformation of the plate (Figures 6A,B). Constructs instrumented with stainless steel and titanium exhibited different behaviour in terms of stiffness (443 ± 55 N/mm and 192 ± 20 N/mm), yield load (910 ± 93 N and 480 ± 16 N), and maximum load (1,479 ± 133 N and 667 ± 22 N), respectively.

### 3.2 Implant material properties

**FIGURE 7:**
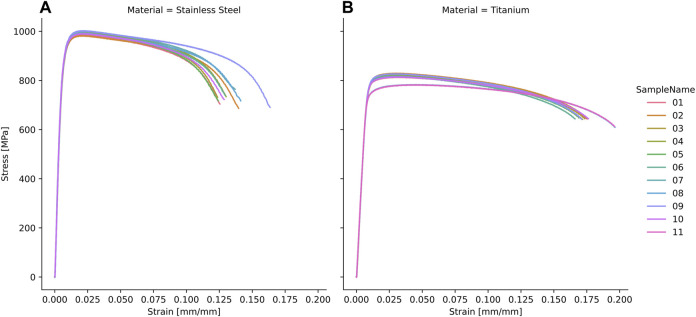
Stress-strain curves of the tested stainless steel **(A)** and titanium **(B)** samples.

All dog bone shaped samples failed within the midsection during uniaxial tensile testing. The stress-strain curves (Figure 7) and all resulting properties including Young’s modulus, yield stress and ultimate strain revealed high consistency and repeatability (Table 2). The experimentally determined material properties corresponded well to the data provided by the implant manufacturer (Disegi, 2008a; Disegi, 2008b) but differed from literature-based values for both materials (Table 2).

**TABLE 2:** Material properties based on reported values in the literature (Mat_Lit_), as reported from the manufacturer (Mat_Man_), and based on the current tensile tests (Mat_Exp_).

	Titanium (3.7065)			Stainless steel (1.4441)		
	Mat_Lit_ (Chen et al., 2010; Zhou et al., 2017; Antoniac et al., 2019; Beirami et al., 2021; Takahashi et al., 2021)	Mat_Man_ (Disegi, 2008b)	Mat_Exp_	Mat_Lit_ (Chen et al., 2010; Mohandes et al., 2021)	Mat_Man_ (Disegi, 2008a)	Mat_Exp_
Young’s modulus [GPa]	110	104	104.6 ± 1.1	200	186.4	183.8 ± 7.8
Yield Strength Rp 0.2 [MPa]	600	≥520	761.3 ± 16.4	690	≥690	874.7 ± 8.6
Ultimate Tensile Strength [MPa]	n.a.	≥680	813.5 ± 16.0	n.a.	≥860–1,100	994.0 ± 6.4
Elongation at failure [%]	n.a.	≥10	17.6 ± 1.0	n.a.	≥12	13.4 ± 1.2

### 3.3 Validation of subject-specific FE models of *in vitro* study

**FIGURE 8:**
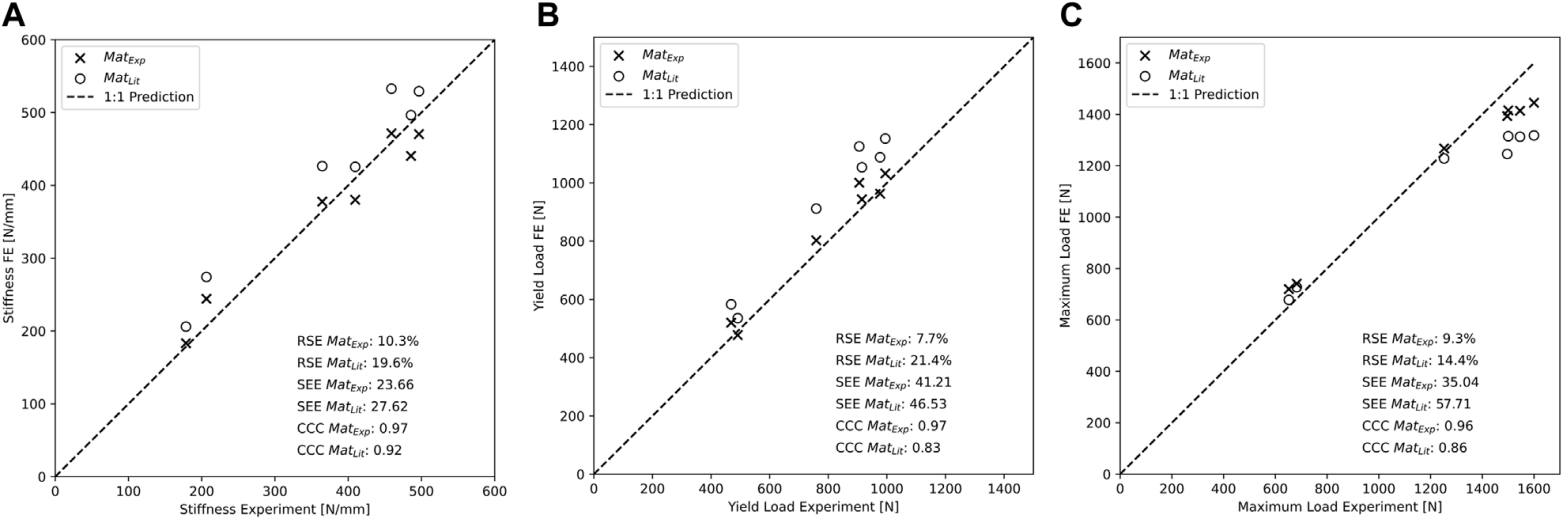
Construct stiffness **(A)**, yield load **(B)**, and maximum load **(C)** are better predicted by the specimen-specific FE models using the experimentally determined material properties versus literature-based properties.

Specimen-specific FE models with experiment-based implant material properties (Mat_Exp_) were able to accurately predict experimental construct stiffness (CCC = 0.97, SEE = 23.66 N/mm, RSE = 10.3%, Figure 8A, Table 3), yield load (CCC = 0.97, SEE = 41.21 N, RSE = 7.7%, Figure 8B), and maximum load (CCC = 0.96, SEE = 35.04 N, RSE = 9.3%, Figure 8C). The FE prediction accuracy was lower when using the literature-based material properties (Mat_Lit_) for construct stiffness (CCC = 0.92, SEE = 27.62 N/mm, RSE = 19.6%, Figure 8A), yield load (CCC = 0.83, SEE = 46.53 N, RSE = 21.4%, Figure 8B), and maximum load (CCC = 0.86, SEE = 57.71 N, RSE = 14.4%, Figure 8C), as summarized in Table 3.

**TABLE 3:** Summary of the prediction capabilities of the FE models using material properties based on the reported values in the literature (Lit) and on the tensile testing experiments (Exp), described by the concordance correlation coefficient (CCC), standard error of estimate (SEE) and relative standard error (RSE).

	CCC		SEE		RSE	
	Exp	Lit	Exp	Lit	Exp	Lit
Stiffness	0.97	0.92	23.66 [N/mm]	27.62 [N/mm]	10.3%	19.6%
Yield load	0.97	0.83	41.21 [N]	46.53 [N]	7.7%	21.4%
Maximum load	0.96	0.86	35.04 [N]	57.71 [N]	9.3%	14.4%

## 4 Discussion

FE simulations are widely used in orthopaedics, mainly to analyse the biomechanical behaviour and stability of bone-implant constructs (Taylor and Prendergast, 2015; Lewis et al., 2021). However, the models often lack validation and only consider the elastic properties of the implant (Lewis et al., 2021). The validation approach employed in this study, involving the development of an elasto-plastic FE model with experimentally determined implant properties and its validation using *in vitro* cadaveric construct test, has demonstrated promising results in accurately predicting the post-plastic behaviour of osteosynthesis constructs. By incorporating experimentally determined values of elastic and plastic properties of locking plates, the models’ predictive capabilities have been enhanced compared to literature-base properties, offering a more accurate prediction of the mechanical behaviour of the fracture fixation construct.

The FE simulations were able to predict the construct stiffness, yield load, and maximum load of the *in vitro* cadaveric experiments highly accurately, proving the validity of the modelling approach and thus confirming the initial main hypothesis. Kopec et al. investigated broken implants and hypothesized that these failed most likely due to mechanical overloading resulting from either repetitive or excessive limb loading (Kopec et al., 2021). They concluded that either the implant design was not fully suited to withstand the mechanical forces and moments, or the underlying physiological activity was excessive. Besides fatigue plate fractures, plastic implant deformation is another failure type seen in patients with good bone stock quality, leading to hardware failure if the applied loads exceed the implant capacity, especially in load bearing fixations of unstable comminuted fractures where the implant entirely carries the load (Perren et al., 2014). Similarly, the osteotomy gap sizes in the present study were large enough to avoid interfragmentary far cortex contact of the bone, thus the construct behaviour was mainly determined by the implant bending. In such load bearing scenarios, knowledge of the accurate implant material properties is crucial to accurately model the behaviour of osteosynthesis constructs when predicting failure. Taylor et al. described the general difficulty of collecting the material properties of all materials and brands (Taylor and Prendergast, 2015), thus literature values are often used for modelling. The importance of implant material properties might change when investigating different failure modes related rather to the bone component of the fixation construct, such as screw pull-out, cut-out, or perforation, or when analysing construct stiffness in the elastic region only. However, this study provided evidence for the importance of determining and using accurate material properties as the results were highly sensitive to the implemented values.

Implant material properties evaluated via uniaxial tensile testing of dog bone shaped samples were highly reproducible. When comparing the results to the used values in the literature, differences could be observed. Others used solely elastic properties, usually ranging between 195–210 GPa for stainless steel (Chen et al., 2010; Antoniac et al., 2019; Beirami et al., 2021; Takahashi et al., 2021) and 96–110 GPa for titanium alloy (Chen et al., 2010; Antoniac et al., 2019) LCPs. For implant grade stainless steel (1.4441), the Young’s modulus is often reported higher in literature as 200 GPa or 210 GPa versus the 183.8 GPa found in this study. Only a few previous studies on implant-related FE modelling investigated the post-elastic behaviour of osteosynthesis plates and thus, literature-based values are scarce. Reports by Zhou et al. and Mohandes et al. used yield stresses for titanium and stainless steel that corresponded with the minimum yield strength provided by the manufacturer but were lower compared to our results (Disegi, 2008a; Disegi, 2008b; Zhou et al., 2017; Mohandes et al., 2021). Simulating properties with underestimated values may suffice for worst-case analyses but not for accurate modelling of construct behaviour. When using literature-based implant material properties in our analyses, the concordance correlation coefficient, the standard error of estimate, and the relative standard error were lower compared to the results with experimental-based values, suggesting that the construct behaviour could be predicted with higher accuracy with the latter approach. Moreover, the measured properties were well in line with specifications of implant manufactures for stainless steel and titanium alloy LCPs (Disegi, 2008a; Disegi, 2008b) that were found to be very close to the values obtained by tensile testing, further underlining the accuracy of the tests.

Several limitations apply to the current study. The evaluated implant material properties were based on a single raw material profile bar of stainless steel or titanium. Even though this resulted in a small variance between the samples, the properties might vary between different suppliers and manufacturing series. To accurately determine the range of the different properties, multiple series from different suppliers would need to be analysed. Furthermore, the cadaveric experiments consisted of seven specimens of which only two were instrumented with titanium plates. However, the aim of the study was not to compare the differences in construct behaviour between the two implant materials but to validate the FE model. The fixation constructs were tested in compression-bending only and it was not investigated whether the behaviour in other loading modes could be predicted accurately. No post-elastic material behavior of the bone was incorporated in the simulations. However, no bone damage was observed around the screws after testing. Furthermore, a tied interface between screw and bone without the existence of press-fit forces was assumed. However, MacLeod et al. have demonstrated that the load-deformation characteristics of a construct exhibit minimal sensitivity to the utilization of a tied interface between the screw and bone, thereby justifying the chosen modeling approach (MacLeod et al., 2012). It is important to acknowledge that further studies and refinements to the FE model would be necessary to account for a broader range of implant failures such as fatigue failure caused by healing disturbances and incorporating musculoskeletal models to model more realistic *in vivo* loading before translating the models to the clinics. Nonetheless, this validation will contribute to the development of more reliable and effective treatment strategies for bone fracture management.

In conclusion, FE models of cadaveric ovine tibia shaft fracture fixations using relative stability via bridge plating with experimentally determined elasto-plastic material properties of stainless steel and titanium LCPs were able to predict construct stiffness and failure with high accuracy. The findings that the use of these properties significantly enhanced the predictive performance of the FE model compared to literature-based values emphasize the importance of carefully determining the appropriate elasto-plastic implant material properties either from the manufacturer or by experimental testing. Consequently, these models can be used to determine the forces needed to cause plastic deformations—i.e., overloading failure—in osteosynthesis constructs. In the future, these validated models could be used to predict the level of loads that cause the observed plate deformations *in vivo* or guide preoperative planning and postoperative rehabilitation protocols. However, to maximize prediction accuracy, potential errors at each step of the modeling workflow should be eliminated in an isolated manner, as it was presented here for the implant material properties.

## Data Availability

The raw data supporting the conclusion of this article will be made available by the authors, without undue reservation.
